# Molecular and Epigenetic Mechanisms of MLL in Human Leukemogenesis

**DOI:** 10.3390/cancers4030904

**Published:** 2012-09-10

**Authors:** Erica Ballabio, Thomas A. Milne

**Affiliations:** MRC Molecular Haematology Unit, Weatherall Institute of Molecular Medicine, John Radcliffe Hospital Headington, Oxford OX3 9DS, UK; E-Mails: erica.ballabio@imm.ox.ac.uk (E.B.)

**Keywords:** MLL, epigenetics, leukemia, super elongation complex, transcription, microRNAs

## Abstract

Epigenetics is often defined as the study of heritable changes in gene expression or chromosome stability that don’t alter the underlying DNA sequence. Epigenetic changes are established through multiple mechanisms that include DNA methylation, non-coding RNAs and the covalent modification of specific residues on histone proteins. It is becoming clear not only that aberrant epigenetic changes are common in many human diseases such as leukemia, but that these changes by their very nature are malleable, and thus are amenable to treatment. Epigenetic based therapies have so far focused on the use of histone deacetylase (HDAC) inhibitors and DNA methyltransferase inhibitors, which tend to have more general and widespread effects on gene regulation in the cell. However, if a unique molecular pathway can be identified, diseases caused by epigenetic mechanisms are excellent candidates for the development of more targeted therapies that focus on specific gene targets, individual binding domains, or specific enzymatic activities. Designing effective targeted therapies depends on a clear understanding of the role of epigenetic mutations during disease progression. The Mixed Lineage Leukemia (MLL) protein is an example of a developmentally important protein that controls the epigenetic activation of gene targets in part by methylating histone 3 on lysine 4. MLL is required for normal development, but is also mutated in a subset of aggressive human leukemias and thus provides a useful model for studying the link between epigenetic cell memory and human disease. The most common MLL mutations are chromosome translocations that fuse the *MLL *gene in frame with partner genes creating novel fusion proteins. In this review, we summarize recent work that argues MLL fusion proteins could function through a single molecular pathway, but we also highlight important data that suggests instead that multiple independent mechanisms underlie MLL mediated leukemogenesis.

## 1. Introduction

Genome wide association studies provide a wealth of information on cancer driver mutations as well as revealing the incredible range of genetic diversity in human cancers [1]. However, epigenetic changes could account for the bulk of variation in cancer and thus may be an even more important determinant of clonal evolution and disease progression [1]. Epigenetics is described as the study of heritable changes in gene expression that are not due to modifications in the DNA sequence. These changes include DNA methylation, small, non-coding RNAs and histone modifications [2,3]. Epigenetic changes play a crucial role in the regulation of gene expression and aberrations in the epigenetic program can induce alterations in cellular proliferation, apoptosis and differentiation [2]. Aberrant epigenetic changes are correlated with different cancer subtypes and prognoses [2,4,5,6,7,8,9,10,11,12,13]. A clear understanding of epigenetic alterations on the molecular level can provide crucial information for the development of targeted therapies that focus on subsets of gene targets, small molecular inhibitors of individual binding domains, or specific enzymatic activities [14,15,16].

Histone proteins are considered to be one of the carriers of epigenetic information and are complexed with DNA to produce a protein/DNA structure called chromatin. The basic subunit of chromatin is the nucleosome and each nucleosome is composed of an H3/H4 tetramer and two H2A/H2B dimers [17,18]. Post-translational modifications of histone proteins are considered to be one of the epigenetic mechanisms that multicellular organisms use in order to guarantee tight spatial and temporal expression of key genes during development and differentiation [19,20,21]. These modifications include “marks” such as phosphorylation (P), acetylation (Ac), methylation (Me, which can be added as a mono (1), di (2) or tri (3) methyl mark) and ubiquitination (Ub) which function by recruiting and/or stabilizing specific effector proteins (also referred to as “reader” proteins) [22]. Histone marks can also be used in genome wide studies as a way of demarcating different functional regions of the genome [23,24,25]. Specifically, H3K4Me1 and H3K27Ac together mark active gene enhancers while H3K4Me3 specifically marks “poised” and active promoters, H3K79Me2 and H3K36Me3 together mark the coding regions of actively elongating genes and H3K27Me3 marks repressed genes [23,24,25,26,27,28]. Although the heritability of histone modifications themselves is not completely established, a great number of epigenetic cell memory proteins that have been implicated in human disease have also turned out to be enzymes that are involved in “writing”, “erasing” or “reading” histone modifications [4,16,22].

The Mixed Lineage Leukemia protein (MLL) is required for the epigenetic maintenance of gene activation during development [29,30,31] and is also mutated in a subset of aggressive acute leukemias [32]. MLL maintains gene activation in part by methylating histone 3 on lysine 4 [33,34]. The most common leukemogenic *MLL *mutations are chromosome translocations that fuse the *N*-terminus of the *MLL *gene in-frame with any of more than 60 different partner genes producing novel MLL fusion proteins (MLL-FPs) [32,35]. MLL-FP leukemias have very few additional genetic mutations [36,37,38] suggesting that the MLL-FP mutation alone is sufficient for initiating leukemogenesis. Interestingly, some of the molecular data assembled to identify and analyze recent epigenetic inhibitors has come from the analysis of MLL-FP leukemias [14,15,16]. This suggests that MLL provides a useful model for studying the link between epigenetic cell memory and human disease and may provide information on pathways and targets that are more generally applicable to a wider range of different cancers.

## 2. MLL in Normal Hematopoiesis

*MLL *was originally identified as a functional ortholog of the *trithorax* gene in *Drosophila* [39,40,41]. Wild type MLL is crucial for maintaining the activation of important genes such as the *Homeobox* (*Hox*) genes in embryogenesis [42,43,44], hematopoiesis [29,31] and neurogenesis [30]. *Hox* genes are a group of highly conserved genes important for the regulation of gene expression and axial patterning during development [45]. Highlighting the role of MLL as an epigenetic cell memory protein, *Mll1 *mutant mice display normal early *Hox *gene expression patterns but then lose maintenance of expression as development proceeds [43]. It is worth noting that *trithorax* mutants in *Drosophila* display similar *Hox* gene expression maintenance defects [46].

Mice heterozygous for *Mll1 *show retarded growth, hematopoietic abnormalities and bidirectional homeotic transformations of the axial skeleton, typical of *Hox* misexpression [44]. Furthermore, mice expressing a deleted form of *Mll1 *lacking the SET (Su (var), Enhancer of Zeste and Trithorax) domain (also see Section 3 below) are fertile and viable although they display developmental skeletal defects and abnormal transcription levels of several *Hox *loci during development [47]. *Mll1 *null mice die between embryonic day 12.5 and 16.5. Expression of *Hox* genes is initiated in these mice but then decreases once the function of *Mll1 *becomes necessary for their maintenance [43,44]. They exhibit defects in hematopoiesis in the fetal liver due to decreased expression of *HoxA4*, *HoxA7*, *HoxA9*, and *HoxA10 *leading to a severe reduction in the number of long term and short-term hematopoietic stem cells (HSCs). The remaining HSCs are able to support a limited expansion, however they can’t contribute to hematopoiesis when transplanted into irradiated mice, suggesting a defect in their self-renewal capacity [31]. Interestingly, in an ES cell model, the block in hematopoiesis can be rescued by reintroducing individual *Hox* genes [48]. Although it is difficult to say from these experiments whether *Hox* gene expression alone can compensate for the loss of *Mll1* in murine hematopoiesis, this data does suggest the possibility that the essential role played by *Mll1 *is in fact linked to deregulation of *Hox* genes [48]. Jude *et al*. showed that deletion of *Mll1 *in adult mice leads to fatal bone marrow failure within three weeks due to reduced proliferation and reduced response to cytokine-induced cell-cycle entry of myelo-erythroid progenitor cells and depletion of quiescent HSCs, indicating that *MLL *is also important in adult hematopoiesis [29].

## 3. Activity of the Wild Type MLL Protein Complex

In mammals, MLL belongs to a family of H3K4 methyltransferases (KMTs) that also includes SET domain containing 1A (SETD1A), SETD1B, MLL2, MLL3, MLL4 and MLL5 (Summarized in Table 1 and [49]). Despite having similar enzymatic activities, these H3K4Me writer proteins exhibit different phenotypes and are important for the regulation of different gene targets [44,50,51,52,53,54,55,56,57,58,59]. It is beyond the scope of this review to discuss the different MLL family protein complexes, but determining whether or not they bind to unique target genes or have unique regulatory functions at the same target genes remains an important area of study.

**Table 1:** MLL family names.

NCBI Name	Aliases (commonly used aliases in bold)	Human Chromosome Position	Important References
MLL	**MLL1**, HRX, TRX1, ALL-1, CXXC7, HTRX1, MLL1A, MLL/GAS7, TET1-MLL, KMT2A	11q23	[39,40,41,60]
MLL2	**ALR**, KMS, **MLL4**, AAD10, KABUK1, TNRC21, CAGL114, KMT2B, KMT2D	12q13	[61] Note: this is the MLL that is mutated in Kabuki syndrome [62], sometimes MLL4 (below) is mistakenly referenced
MLL3	**HALR**, KMT2C	7q36.1	[63,64]
MLL4	**MLL2**, HRX2, TRX2, WBP7, KMT2D	19q13.1	Originally called MLL2 in [65,66], renamed MLL4 in [63] but still commonly referred to as MLL2 in [51,54,67,68,69]
MLL5	HDCMC04P, KMT2E	7q22.1	[70]
SETD1A	**SET1A**, SET1, KMT2F	16p11.2	[71]
SETD1B	**SET1B**, KMT2G	12q24.31	[72]

**Figure 1:**
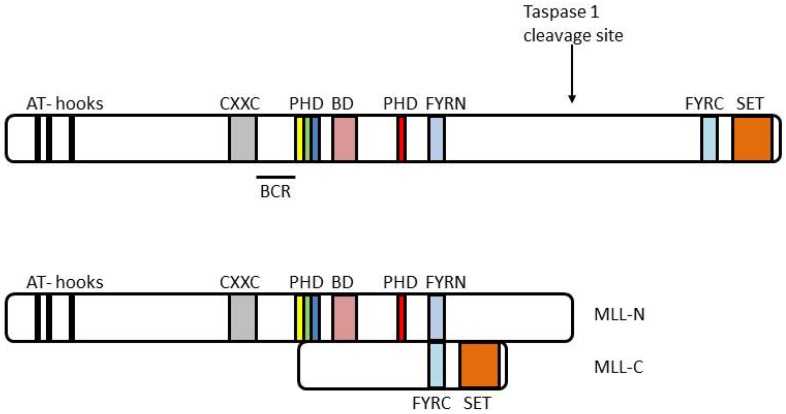
MLL protein structure. MLL has 3 HMG-like AT hooks (black bars) that bind AT rich DNA, a CXXC domain (grey bar) that binds unmethylated CpG DNA, four PHD (Plant Homeo Domain) fingers (yellow, green blue and red bars) that mediate interactions with several proteins; an atypical bromodomain (purple bar), FYRN and FYRC domains (light blue bars) and a C terminal SET domain (orange bar) that methylates histone H3 on lysine 4. Wild type MLL is cleaved by taspase 1 into two fragments: MLL-N and MLL-C. These fragments dimerize to form a stable complex. BCR = breakpoint common region. Adapted from [69].

*MLL *encodes a 3,969 amino-acid DNA-binding protein which contains several domains including two AT-hooks that can bind AT rich DNA [73], a CXXC domain that binds unmethylated CpG rich DNA [74,75,76,77] and the polymerase associated factor 1 (PAF1) elongation complex (PAF1C) [69,78], four PHD fingers that interact with cyclophilin 33 (CyP33) [79,80,81,82,83], the H3K4Me2/3 mark [69,82,84] and the Elongin B/C-Cullin-SOCS box protein (ECS^ASB^) E3 ubiquitin ligase complex [85], an atypical bromodomain [82], and a C-terminal SET domain that catalyzes H3K4 KMT activity ([33,34] and Figure 1). For the sake of brevity, this review will focus on a few key MLL complex members, but Table 2 presents a summary of proteins shown to bind directly to MLL.

**Table 2:** MLL protein complex components.

NCBI Name	Aliases (commonly used aliases in bold)	Human Chromosome Position	MLL interaction site	Evidence for a direct interaction with MLL	Structural data supporting interaction
MEN1	**Menin**, MEAI, SCG2	11q13	*N* terminus	[86]	[87,88,89,90]
PSIP1	p52, p75, PAIP, DFS70, **LEDGF**, PSIP2	9p22.3	*N* terminus	[91]	[90]
PAF1		19q13.1	CXXC region	[69,78]	No data
CTR9	SH2BP1, TSBP, p150, p150TSP	11p15.3	CXXC region	[78]	No data
BMI-1	RNF51	10p11.23	CXXC region	[83]	No data
ELAC2	ELC2, **HPC2**	17p11.2	CXXC region	[83]	No data
CTBP1	CtBP	4p16	CXXC region	[83]	No data
HDAC1	HD1, RPD3, RPD3L1	1p34	CXXC region	[83]	No data
PPIE	CYP-33, **CYP33**	1p32	PHD finger 3	[79]	[82]
ASB2		14q31-q32	PHD fingers	[85]	No data
HCFC1	CFF, HCF-1, **HCF1**, HFC1, VCAF	Xq28	adjacent to BD	[92]	No data
HCFC2	HCF-2, HCF2	12q23.3	adjacent to BD	[92]	No data
CREBBP	**CBP**, RSTS, KAT3A	16p13.3	MLL-C	[93]	No data
KAT8	**MOF**, hMOF, **MYST1**	16p11.2	MLL-C	[94]	No data
WDR5	SWD3	9q34	SET domain	[95,96]	[97,98,99,100,101,102,103,104,105,106]
RBBP5	RbBP5, SWD1	1q32	SET domain	[95,96,107]	[104,105,108]
ASH2L *	ASH2, ASH2L1, ASH2L2, Bre2	8p11.2	SET domain	[95,96,107]	[105,108,109]
DPY30 *	DPY-30, Saf19	2p22.3	SET domain	[96,110]	[111]

* Although ASH2L and DPY30 do not interact directly with MLL, they are included in Table 2 because they are important components of the SET domain core complex.

**Figure 2:**
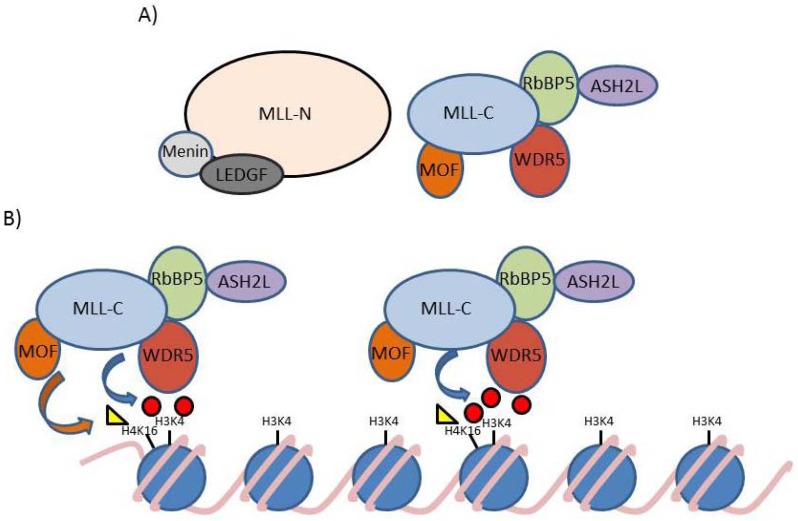
MLL multiprotein complex. (**A**) MLL is present in the cell as part of a large protein complex. MLL-C is associated with the WDR5, RBBP5, ASH2L and MOF proteins. MLL-N interacts with a wide range of different proteins, for simplicity only menin and LEDGF are shown; (**B**) The model proposed by Wysocka *et al*. and Dou *et al*.: WDR5 recruits the MLL complex to H3K4me2 allowing the addition of another methyl group to histone H3 lysine 4 and acetyl group to histone H4 lysine 16. Additional MLL complexes are then recruited to target genes or existing MLL complexes are transferred to the next nucleosome [95,114]. The yellow triangle represents an acetyl mark, while red circles represent methyl marks. Figure adapted from [114].

Taspase 1 mediates the cleavage of the MLL protein generating 320 kDa *N*-terminal (MLL-N) and 180 kDa *C*-terminal (MLL-C) fragments which then together dimerize in a large molecular weight complex [34,92,94,112,113]. The MLL-C core complex that mediates H3K4 methylation consists of WD repeat-containing protein 5 (WDR5), ash2 (absent, small, or homeotic)-like (Drosophila) (ASH2L) and Retinoblastoma binding protein 5 (RBBP5) RBBP5 [95] (Figure 2A). WDR5 mediates the binding of the MLL complex to H3K4me2 and is important for the conversion of di- to tri-methyl at lysine 4 [114] while RBBP5 stabilizes the interaction between MLL-C and WDR5 as well as ASH2L [92,95]. The MLL-C complex also interacts with the histone acetyltransferase males absent on first (MOF aka MYST1, see Table 2) which acetylates H4K16 [94] and the CREB binding protein (CBP) that acetylates H3 and is important for the transcriptional activation of specific target genes [93]. According to the model proposed by Wysocka *et al*. and Dou *et al*., once methylation is initiated, WDR5 recruits the MLL complex to H3K4me2 allowing the addition of another methyl group at K4 (H3K4me3) and acetylation of H4K16. Additional MLL complexes are then recruited or MLL complexes are transferred to the next nucleosome through the binding of WDR5 to H3K4me2 (Figure 2B) [95,114].

As noted above and in Table 2, the MLL-N portion binds to a large number of different proteins, we briefly discuss here two key ones. The *N*-terminus of MLL binds to the menin (MEN1) and lens epthelium-derived growth factor (LEDGF) proteins [86,87,88,89,91,92,115] (Figure 2A). Menin is a tumor suppressor protein whose loss causes multiple endocrine neoplasia type 1, an autosomal dominant familial cancer syndrome [116]. Menin and LEDGF were initially thought to be essential for the recruitment of wild type MLL and MLL-FPs to gene targets *in vivo* [90,91,115,117,118,119]. Although the presence of menin tends to be associated with increased gene activation and increased levels of MLL protein [68,115,118,119], its actual role appears to be more complex than a simple MLL recruitment mechanism. The roles of menin, LEDGF and other MLL-N protein/domain interactions in recruitment are discussed in more detail in sections 5 and 9 below.

## 4. MLL and Leukemia

Rearrangements of the *MLL *gene are associated with acute myeloid (AML), lymphoid (ALL), and biphenotypic or mixed lineage leukemias. *MLL *rearrangements are detected in over 70% of all infant leukemias and in 5–10% of childhood and adult ALL or AML cases; they are also a common cause of chemotherapy related secondary leukemias ([120] and reviewed in [121,122]). As a general group, the genetics of acute leukemias are often complex and carry a wide range of different somatic mutations combined with complex gene expression patterns. Conversely, MLL-FP leukemias represent a distinct class of acute leukemias that are relatively simple from a genetic point of view.

There are two general groupings of *MLL *rearrangements: (1) *MLL *gene specific mutations that include small internal deletions, partial tandem duplications (*MLL-PTD*) or *MLL *whole gene duplications; and (2) large, cytologically visible chromosome translocations that fuse the 5' end of the *MLL *gene in-frame with over 60 different partner genes producing novel MLL-FPs [35]. *MLL-PTD *is associated with 5–10% of AML with normal karyotype and 25% of these patients also carry a *FLT3* (*FMS-like tyrosine kinase 3*) internal tandem duplication (*FLT3-ITD*) mutation, suggesting that *MLL-PTD *requires cooperating mutations to produce AML [123,124]. A recent study using an animal model reported that *MLL-PTD* directly contributes to AML when present with another mutation such as *FLT3-ITD * [125]. Zorko *et al*. have shown that while animals expressing either *Mll^PTD/WT^:Flt3^ITD/WT^* are not able to develop leukemia, double knock-in mice are capable of recapitulating the human disease [125].

Conversely, MLL-FPs alone can directly cause aggressive acute leukemias in mouse model systems as well as in Xenograft assays [12,126,127,128,129,130,131,132,133,134,135,136,137,138,139]. In human patients, the prognosis for leukemias containing MLL-FPs varies somewhat with the different fusion partners but is generally quite poor ([140,141], reviewed in [122] and in [142]). There are two general classes of MLL-FPs, nuclear FPs and cytoplasmic FPs. Cytoplasmic FPs are much more rare and are thought to function by adding dimerization domains to MLL-N [135,138,143,144]. Around 80–90% of all *MLL* gene translocations are accounted for by fusions with the *AF4*, *AF9*, *ENL*, *ELL*, *AF10* or *AF6* genes [145], while the remaining 59 different fusion partners, most of which were identified in only single patients, account for 10–20% of MLL-FPs [35]. In this review, we will specifically focus on the function of the six most common MLL-FPs and discuss recent work that suggests that this subset share a common biochemical mechanism.

Rare among *MLL* leukemias, the reciprocal fusion gene from the t(4;11)(q21;q23) chromosomal translocation is expressed producing both MLL-AF4 and AF4-MLL proteins [146,147]. Although co-expression of both MLL-AF4 along with AF4-MLL most closely recapitulates the human disease phenotype [146], knocking down the MLL-AF4 protein is sufficient to disrupt the leukemic growth of a t(4;11) patient cell line [148] suggesting that targeting MLL-AF4 activity alone may be sufficient for disrupting t(4;11) leukemogenesis. Up to 80% of all t(4;11) leukemias express both AF4-MLL and MLL-AF4, with the remaining 20% expressing MLL-AF4 alone [149]. In this review, we will focus mainly on the function of the common MLL-FPs and will not explore the function of the AF4-MLL protein in depth, but it is likely that this protein will turn out to be a key player in t(4;11) leukemias.

Two non-exclusive models have been proposed to explain the relative aggressiveness of MLL-FP leukemias including (a) that MLL-FPs increase mutation rates in cells and (b) that MLL-FPs hijack multiple cellular growth pathways [150].

Favoring the first model is the finding that MLL has a key role in the DNA damage response (DDR) pathway and MLL-FPs cause compromised S phase checkpoints and chromatid errors [151]. Normally, MLL is phosphorylated by ATR (ataxia telangiectasia and Rad-3-related) protein in response to DNA damage during S phase. Phosphorylation of MLL prevents its degradation by the Skp2 (S-phase kinase-associated protein 2) protein and promotes its accumulation on chromatin, ultimately leading to a delay in DNA replication fork assembly [151]. However, the presence of MLL-FPs impairs the phosphorylation of the wild type protein and its stabilization on the damaged DNA, thus compromising the S-phase checkpoint and allowing accumulation of mutations [151]. Moreover, a recent study has highlighted the importance of a DNA damage response signaling pathway in an MLL-ENL mouse model [152]. Induced expression of the fusion protein causes myeloproliferation with the potential to develop full acute leukemia. This in turn activates the DNA damage response leading to an arrest in proliferation and eventual senescence. Positive selection for those cells able to eliminate or bypass this response allows for the transition from a pre-leukemic state, with myeloproliferative characteristics, to a fully developed leukemia. Activation of a DDR pathway was also described in clinical samples of human MLL leukemic patients, although the downstream effector pathways were reported to be attenuated suggesting that the DDR has to be neutralized in order to progress to full disease [152].

On the other hand, supporting the hypothesis that MLL-FPs alone are sufficient for the leukemic transformation of the target cells, high resolution SNP array analyses and genome wide sequencing in t(4;11) leukemias suggest they contain very few additional cooperating mutations [36,37,38]. Also, MLL-FPs alone are often capable of rapidly producing acute leukemias in mice [12,126,127,128,129,130,131,132,133,134,135,136,137,138,139]. This indicates that the expression of MLL-FPs is sufficient for the promotion of leukemogenesis, likely through the epigenetic activation of key master regulatory factors that set up gene expression networks responsible for cell growth and proliferation.

Although much progress has been made in elucidating the molecular basis underlying MLL-FP mediated leukemogenesis, attempts to find common mechanisms have been complicated by the heterogeneous nature of these leukemias. For instance, *MLL-AF4* fusions are predominantly found in ALL (although also detected in biphenotypic ALL, therapy related AML and rare cases of AML), *MLL-ELL*, *MLL-AF10* and *MLL-AF6* fusions are predominantly present in AML, while *MLL-AF9* and *MLL-ENL* are found in both AML and ALL (although rare in adult ALL, reviewed in [121,122,142]). 

Different MLL-FPs are often associated with different prognoses, even when they produce similar leukemias [142]. Elegant experiments from the Rabbitts laboratory have demonstrated that the fusion partner has an active role in determining the resulting leukemia [153,154,155,156,157]. Specifically, they used an *in vivo* Cre-loxP mediated recombination system for inducing *MLL* chromosome translocations in different mouse cell lineages. They found that *MLL-AF9* translocations could only cause AML and had no capability to produce ALL even when induced in T cells, *MLL-ENL *could cause both AML or T-ALL (but not B cell ALL) depending on whether it was expressed in a more primitive or a more differentiated cell, and *MLL-AF4* could only produce B cell lymphomas even when induced in T cells [153,154,155,156,157].

An important conclusion from this work is that it is the combination of the specific fusion partner and the cell type where the fusion is expressed that determines the resulting leukemia. So how does the fusion partner influence the leukemic outcome? The fact that different MLL-FPs can be expressed in the same cell type and have different phenotypic outcomes suggests that different MLL-FPs either regulate different target genes, or they have unique functions at a similar set of target genes. This produces the following two key questions:

(1) Which key downstream gene targets are essential for MLL-FP mediated leukemogenesis?
(2) How do different MLL-FPs control epigenetic gene regulation on a molecular level?

## 5. Recruitment of MLL and MLL-FPs to Gene Targets

There are several lines of evidence suggesting that recruitment and stable binding of MLL and MLL-FPs are key to keeping target genes such as *HOXA9* active. MLL binding and *HOXA9* expression are both very dynamic during hematopoietic differentiation and are tightly linked [13,20,48,158]. Binding of MLL is also an essential step for *HOXA9* activation in mouse embryonic fibroblasts (MEF) cells [13,20,69,94,95] and MLL-FP binding is absolutely necessary for *HOXA9* activation in bone marrow cells [13]. As discussed earlier, MLL is rich with chromatin binding domains, all of which could potentially act as reader modules that control recruitment. The concept of “multivalency”, where multiple interactions are required for specific and stable binding [159], has become an increasingly studied mechanism for how chromatin proteins recognize target genes *in vivo * [160,161].

**Figure 3:**
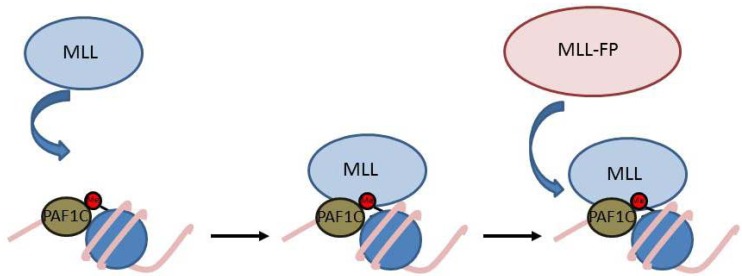
Steps in recruiting MLL and MLL-FPs to the *HOXA9* locus. MLL is recruited to a target gene through interactions with the PAF1 complex and H3K4Me3 which results in increased activation of the locus and a more open chromatin conformation. This allows MLL-FP to bind by interactions with PAF1C and CpG rich DNA. Red circles represent methyl marks.

In a systematic analysis of domain recruitment function, we recently identified a minimal *HOXA9 *recruitment domain that requires the PHD fingers as well as the CXXC domain of wild type MLL [69]. This minimal recruitment domain requires the activity of the third PHD finger of MLL which binds to the H3K4Me2 and Me3 “writer” mark of MLL [69,82], and also requires an interaction between the MLL CXXC domain and the PAF1 elongation complex [69,78]. The PAF1C is made up of six different components which include the already mentioned PAF1 as well as CTR9, RTF1, LEO1, CDC73 and SKI8 [162]. The MLL CXXC domain interacts directly with PAF1 [69,78] and an adjacent region interacts directly with CTR9 [78]. In summary, these results suggest that multivalent interactions control MLL recruitment to *HOXA9 in vivo* (Figure 3). However, since MLL and PAF1C do not completely overlap in the cell [69], this also suggests that MLL recruitment to target genes other than *HOXA9* could be controlled by other mechanisms.

MLL-FPs lack the PHD fingers and thus are recruited to gene targets through a slightly different mechanism. MLL-FPs still interact directly with PAF1C [69,78], but they also bind directly to CpG rich DNA via the CXXC domain and disruption of this interaction completely abolishes recruitment [69]. Interestingly, MLL-AF9 recruitment to *HOXA9* is only possible in the presence of an actively bound wild type MLL complex ([69] and Figure 3). We have no evidence for a direct protein-protein interaction between MLL and MLL-AF9, so we instead favor the idea that binding of the wild type MLL protein to *HOXA9* creates an “open chromatin” conformation which allows MLL-FP binding (Figure 3). It is unclear if MLL-FPs are dependent on wild type MLL for recruitment to all gene targets, or if *HOXA9* is a special case. It is also unclear if the fusion partner itself could contribute to recruitment, or if there is simply a generic recruitment mechanism that operates for all the different MLL-FPs. Much remains to be understood about the recruitment process, which is why this continues to be an important area of inquiry.

## 6. Important MLL-FP Regulatory Targets

### 6.1. Gene Targets

There is conflicting evidence over whether MLL-FPs regulate the same or different target genes. Human leukemias with MLL rearrangements are strongly correlated with expression of the *MEIS1* and *HOXA9* genes [163,164,165,166]. In bone marrow transformation assays and mouse model systems, co-expression of *Meis1* and *HoxA9* together cause aggressive leukemias in mice [167,168] and together can replace the requirement for an active MLL-ENL fusion protein [169]. Importantly, *HoxA9* expression alone (but not *Meis1* alone) can cause leukemia in mice, although with a much longer latency than *HoxA9/Meis1* co-transfected mice [168], and it is required for MLL-ENL mediated bone marrow transformations [170]*. HOXA9* overexpression has long been thought to be a hallmark of MLL-FP leukemias and many human patient samples are dependent on overexpression of *HOXA9* for their continued growth and leukemic potential [171,172]. As well, the vast majority of human patients with MLL-FPs show high levels of *HOXA9* expression [173].

However, other evidence suggests that *HOXA9* may not be a key target in all MLL-FPs: *HoxA9^−/−^* mice are still susceptible to MLL-AF9 mediated leukemogenesis [132] and MLL-GAS7 is capable of transforming *HoxA9^−/−^* bone marrow cells [137]. Furthermore, patients carrying t(4;11) can be equally split into two distinct subsets: those who have *HOXA* gene cluster expression or those that do not [174,175]. Interestingly, patients lacking *HOXA* expression have a worse prognosis and a greater chance for disease relapse [174,175]. Together, these results suggest that *HOXA9* may not be the key target gene in all circumstances.

Several recent papers have identified additional target genes that contribute to MLL-FP leukemogenesis including a central role for *MEIS1* [176], *Eya1*, *Six1* [177], *MEF2C * [178,179], *RUNX2*, *ITF-2* [178], *IGSF4 * [180], *MYB* [181,182], the Wnt pathway [131,139], *CD133* [183], as well as *MYC* target genes in general [184,185,186]. However, it is not clear if any of these gene targets have a role in all MLL-FP leukemias or are instead expressed in only a subset of human cases.

Evidence that different MLL-FPs regulate unique target genes comes from a recent observation that although human patient samples with MLL-AF4, MLL-ENL or MLL-AF9 fusion proteins have overlapping gene expression profiles, there is a subset of gene targets that are distinct for each fusion protein [174]. Additionally, Wang *et al*. have suggested that MLL-FP target genes, at least for MLL-ENL, are a subset of wild-type MLL target genes [177] and a comparison of ChIP-seq data for MLL-AF4 and MLL-AF9 identify few common gene targets [187,188]. Thus the possibility exists that different MLL-FPs may directly regulate unique target genes and this would explain at least some of the heterogeneity observed in MLL-FP leukemias. Future ChIP-seq experiments will likely shed further light on MLL-FP target gene specificity.

Complicating the search for key targets is the leukemic stem cell (LSC) hypothesis which suggests that leukemogenesis is controlled by a small subset of cancer stem cells that control self-renewal and are responsible for relapse after treatment [189]. Although the explicit concept of a rare, phenotypically fixed cancer stem cell remains controversial, it has been suggested that heterogeneity within the cancer stem cell compartment could be a major determinant of clonal evolution and relapse after therapeutic treatment [1]. Recent work from the Vyas laboratory indicates that different subpopulations of LSCs with unique gene expression profiles can exist in the same leukemia, and it seems likely that this level of diversity in gene expression patterns could be controlled by both genetic and as well as epigenetic alterations [190].

Most MLL gene expression analyses have focused on global expression patterns in bulk patient samples, or gene expression patterns in pre-selected retrovirally transduced cell types. Considering that the majority of leukemic cells in a sample do not have LSC potential, it is unclear if the gene expression profiles of the bulk of cells will be truly representative of the LSC core. Retrovirally transduced material is prone to overexpression artifacts [133], suggesting that many studies have not identified the true gene targets necessary for the LSC compartment in human patients. Thus it remains to be determined which downstream targets are the key, direct targets of MLL-FPs that are specifically crucial for maintaining the LSCs in human patients, and if these are shared or unique among different MLL-FPs.

### 6.2. MicroRNA Targets

MicroRNAs (miRs) are a recently discovered class of naturally occurring short non-coding RNA molecules that regulate eukaryotic gene expression through binding to complementary sequences in the 3' UTR of target mRNA. They are commonly aberrantly expressed in many cancers, including hematological malignancies [191]. Patients with AML or ALL can be separated on the basis of their microRNA signature: 27 miRs were found differentially expressed in these 2 groups of patients with *let-7b* and *miR-223* as the most up-regulated and *miR-128a* and *miR-128b* as the most down-regulated [192]. Distinct patterns of microRNA expression are also present in MLL-FP leukemias [193,194,195] with an apparent specific miR signature in AML patients carrying MLL translocations versus other AMLs [196]. An important regulatory target of both MLL and MLL-FPs is *mir-196b*, which is located in a region between *HOXA9 *and *HOXA10 *genes, at chromosome 7p15.2. Recent studies indicate that MLL regulates *mir-196b* expression in a pattern similar to that of the surrounding genes and that MLL-FPs cause its up-regulation [197]. *miR-196b* may play a role in leukemogenesis by stimulating proliferation and inducing a block in differentiation of hematopoietic progenitor cells [197]. Furthermore, expression of MLL-FPs in lineage negative bone marrow cells leads to the overexpression of *mir-196b* and treatment of these cells with an antagomir specific for *mir-196b* causes a decrease in proliferation [197]. Li *et al*. have identified the tumour suppressor gene *Fas* as a direct target of *miR-196b*. Overexpression of *Fas* in mir-196b/MLL-AF9 transduced bone marrow cells significantly delayed the development of leukemia in secondary transplantation, indicating that miR-196b mediated repression of this gene is important for MLL-FP mediated leukemogenesis [198].

Moreover, recent studies have also shown that MLL and in particular MLL-FPs bind to the promoter region of the *miR-17-92* cluster inducing its expression [199]. Over-expression of this microRNA cluster is a common feature in different types of cancers. It has been reported that *miR-17-92* has a role in monocyte, megakaryocyte and B cell development. Forced expression of these miRs causes repression of monocytopoiesis [200] and megakaryocytopoiesis [201] and inhibits the transition from pro- to pre-B cells [202,203]. Ectopic expression of *mir-17-92* increases proliferation in mice bone marrow progenitor cells and contributes to transformation by modulating the expression of important target genes involved in cell cycle, cell death and apoptosis [199]. Recently, Wong *et al*. have identified *CDKN1A* (*p21*), a regulator of the cell cycle, as a direct target of *mir-17-92*. Repression of *p21* would favour cell cycle progression and self-renewal of MLL leukemic cells [204], and may repress the tendency for MLL-FPs to up-regulate the expression of *CDKN1A * [205]. Furthermore, transcription of the *mir-17-92* cluster is directly regulated by MYC [206], which is commonly targeted by MLL-FPs [182,185,186]. These data would suggest a cooperative model where MLL-FPs sustain expression of *MYC* and then together with MYC could activate the *miR-17-92* cluster. Expression of these together could then modulate expression of different target genes in order to maintain proliferation and cell renewal capacity and repress apoptosis.

It’s also possible that down-regulation of specific miRs might maintain overexpression of some MLL-FP target genes. Many of the down-regulated miRs identified in MLL-FP leukemias target important oncogenes that may be critical for leukemogenesis [196]. A good example is m*iR-15a*, one of the most down-regulated microRNAs in MLL-FP leukemias [195,207], which has been known to target the antiapoptotic gene *BCL2 * [208]. *BCL2* is also a common target of MLL-FPs and its expression contributes to MLL-FP leukemogenesis [185]. MLL-FPs could bind to *BCL2* allowing its transcription while the down-regulation of *miR15a* would also support overexpression of this gene. High levels of *BCL2* have potential oncogenic effects in a conditional BCL2 transgenic mouse and may contribute to the pathogenesis of leukemia [208]. *Let-7a *is another microRNA that is down-regulated in MLL-FP leukemias and it has been linked to the RAS family of oncogenes [209]. RAS family proteins are considered to be regulators of different cellular processes [210] and k-Ras cooperates with MLL-FPs to induce leukemia [211,212,213].

Although there is evidence pointing to a common microRNA signature in MLL-FP leukemias, these are quite a heterogeneous group of leukemias and it is conceivable that specific microRNA signatures may instead correlate with individual MLL-FPs. A more comprehensive study designed to differentiate individual MLL-FPs based on their microRNA expression patterns could give us insight into the possible molecular mechanisms driving the different MLL-FPs.

## 7. The MLL-FP Interactome

Despite the fact that there are over 60 different fusion partners, MLL-FPs share many similar features. Breakpoint positions on the MLL protein cluster in a region between the CXXC domain and the PHD fingers ([214,215], Figure 1). This results in a fusion protein lacking the PHD fingers, the bromodomain and the SET domain, but containing the CXXC domain and the *N* terminus of wild type MLL. Thus all MLL-FPs maintain interactions with the previously discussed PAF1C, LEDGF and Menin proteins, and these interactions are all key for MLL-FP mediated leukemogenesis [69,78,86,91].

**Figure 4:**
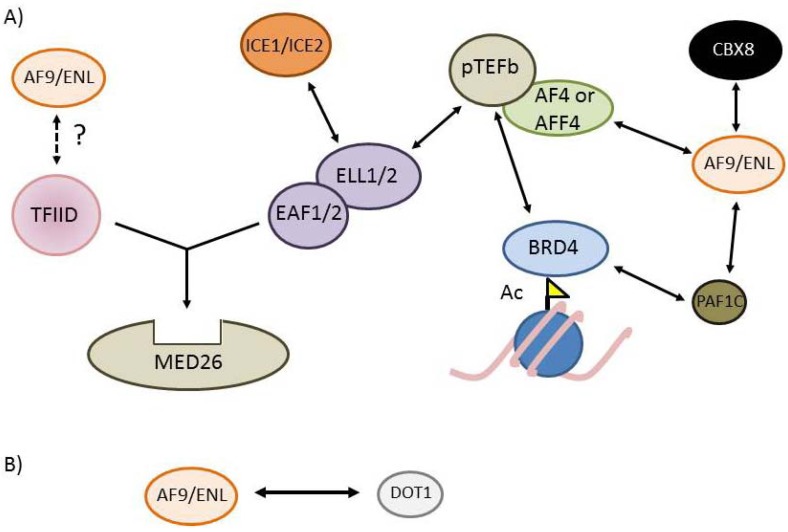
Interactions amongst Super Elongation Complex components (**A**) AF4, AFF4, AF9, ENL, pTEFb, DOT1L, ELL and EAF protein complexes are linked together by a series of different interactions. BRD4 and PAF1C are also linked to the SEC via interactions with pTEFb and AF9/ENL, respectively. Recent data have also suggested the possibility that AF9/ENL might bind TFIID; (**B**) AF9/ENL interacts with the H3K79 methyltransferase DOT1L in a complex that excludes other interacting partners.

**Figure 5:**
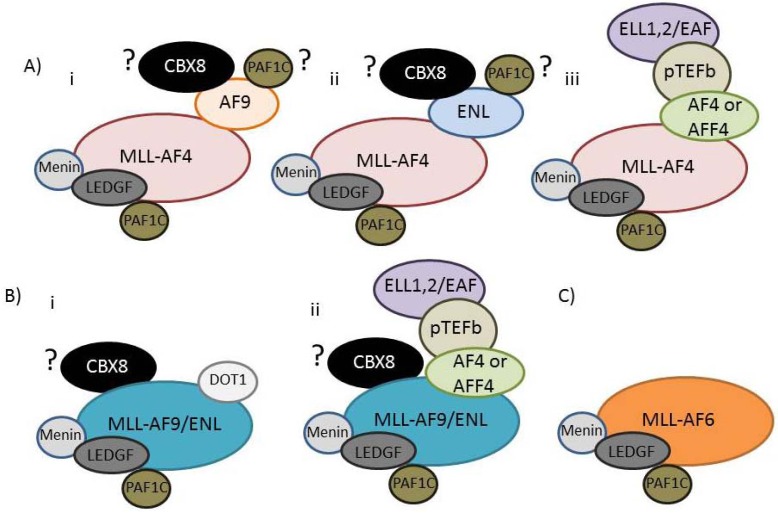
MLL-FPs are present in the cell as part of distinct multiprotein complexes. (**A**) The MLL-AF4 fusion protein can either bind to AF9 (i) or ENL (ii) or AF4/AFF4 (iii). CBX8 and PAF1C may also be able to interact with wild type AF9 and ENL in the context of the MLL-AF4 complexes, or they may be mutually exclusive (the uncertainty is indicated by a “?”); (**B**) MLL-AF9 or MLL-ENL can exist in a complex either with DOT1L or with AF4/AFF4/pTEFb/ELL/EAF. CBX8 may be part of these complexes or may be in a distinct, mutually exclusive complex with MLL-ENL or MLL-AF9; (**C**) MLL-AF6 is the only MLL-FP which doesn’t interact with any SEC component.

Work from multiple labs spanning more than a decade has revealed the presence of multiple shared components in complexes mediated by the fusion proteins themselves (Figure 4 and Figure 5). Using a combination of yeast two hybrid screening, immunopreciptations and GST pulldowns, Erfurth *et al*. initially showed that the wild type AF4 (ALL1-fused gene from chromosome 4 protein) and AF9 (ALL1-fused gene from chromosome 9 protein) proteins directly interact with each other [216]. A synthetic peptide that disrupts the AF4-AF9 interaction *in vivo *causes cell death in MLL-AF4 leukemia cells [217,218], providing strong evidence that fusion protein complexes make key contributions to MLL-FP leukemogenesis. These initial results were extended by the observation that ENL interacts with AF4, AFF4 (AF4/FMR2 family member 4, see Table 3 for additional names), disruptor of telomeric silencing-like (DOT1L, an H3K79 methyltransferase) and, under limited circumstances, AF10 (ALL1-fused gene from chromosome 10 protein) [219,220]. Coupled with an earlier observation that AFF4 interacts with the transcriptional coactivator pTEFb (positive Transcription Elongation Factor b: a dimer of CyclinT1 or T2 and cyclin-dependent kinase 9 (CDK9) that can phosphorylate serine 2 on the RNA pol II C-terminal domain), this suggested that MLL fusion partners could be linked to transcription promoting complexes [221]. Finally, these links were firmly established by work identifying wild type AF4, ENL, AF10 and AF9 as normal components of a complex that contained pTEFb and DOT1L and could promote transcription at target genes [222,223,224].

**Table 3:** MLL interactome names and interactions.

NCBI Name	Aliases (commonly used aliases in bold)	Human Chromosome Position	Established Direct interactions (A question mark indicates a presumed but not fully established interaction)
MLLT3	**AF9**, YEATS3	9p22	DOT1L, AF4, AFF4, CBX8, PAF1, CYCT1
MLLT1	**ENL**, LTG19, YEATS1	19p13.3	DOT1L, AF4, AFF4, CBX8, PAF1, CYCT1?
ELL	C19orf17, **ELL1**, MEN, PPP1R68	19p13.1	EAF1, EAF2,ICE1, ICE2, CYCT1
ELL2		5q15	EAF1, EAF2,ICE1, ICE2, CYCT1
EAF1		3p25.1	ELL, ELL2, MED26, CYCT1
EAF2	BM-040, BM040, TRAITS, U19	3q13.33	ELL, ELL2, MED26, CYCT1
CCNT1(pTEFb)	CCNT, **CYCT1**, HIVE1,Cyclin T1	12q13.11	CDK9, ELL, ELL2, AF4, AFF4, AF9
CDK9(pTEFb)	RP11-228B15.5, C-2k, CDC2L4, CTK1, PITALRE, TAK	9q34.1	CYCT1
AFF1	**AF4**, MLLT2, PBM1	4q21	AF9, ENL, CYCT1
AFF2	FMR2, FMR2P, FRAXE, MRX2, OX19	Xq28	N/A
AFF3	**LAF4**, MLLT2-like	2q11.2-q12	N/A
AFF4	HSPC092, AF5q31, MCEF	5q31	AF9, ENL, CYCT1
DOT1L	**DOT1**, KMT4	19p13.3	ENL, AF9
CBX8	PC3, RC1	17q25.3	ENL, AF9
BRD4	CAP, HUNK1, HUNKI, MCAP	19p13.1	CYCT1, PAF1 complex?
PAF1		19q13.1	ENL, AF9, BRD4?

More recent and extensive analyses of MLL fusion partner complexes have revealed a series of interactions which link together AF4, AFF4, AF9, ENL, pTEFb, DOT1L, ELL (elongation factor RNA polymerase II) and EAF (ELL-associated factor) protein complexes [147,225,226,227,228,229,230], leading to the suggestion that many MLL fusion partner proteins exist together in the cell as a large “super elongation complex” or SEC [226,231]. Interestingly, a common MLL fusion partner in AML, the AF6 protein, does not appear to co-purify with any components of the SEC ([227] and Figure 5C), suggesting that a direct interaction with the SEC is not necessarily essential for MLL-FP mediated leukemogenesis. Exciting recent work has also identified a link between the histone acetyl interacting bromodomain-containing protein 4 (BRD4, a member of the BET family of bromodomain proteins) and the PAF1C and SEC complexes [185], as well as direct interactions between AF9 and ENL with the PAF1C [232], further contributing to the concept of a large fusion partner super complex (Summarized in Figure 4 and Table 3).

One potential difficulty with interpreting these large-scale protein complex purifications is that they fail to differentiate between the existence of one large “super complex”, and the alternate possibility of several smaller sub-complexes that either share overlapping subunits or dynamically interact with each other in the cell [225,227,233]. In a careful analysis of reconstituted minimal complexes, Biswas *et al*. found that the large super complex could be subdivided into smaller, biochemically defined distinct complexes that had some overlapping subunits, as well as several mutually exclusive protein interactions [225]. For instance, although AF4 and AFF4 heterodimerize (and weakly homodimerize) with each other [227], they do not reside in a common complex with AF9 [225]. Also, AF9 and ENL form independent, mutually exclusive complexes with DOT1L that do not contain any other members of the SEC [225,227,232]. Recent work also shows that ENL and AF9 reside in distinct, mutually exclusive SEC complexes, with ENL containing complexes apparently more common in the cell [232]. Similarly, ELL2 containing complexes are more common than ELL containing complexes and degradation of ELL2 impacts the stability of SEC complexes in the cell [234]. The division of the MLL-FP “interactome” into distinct sub-complexes could have implications for understanding how MLL-FPs functionally hijack normal gene regulation processes (see below), including the possibility that different sub-complexes have distinct regulatory targets in the cell [235,236], or the possibility that they impact distinct stages of gene regulation.

Different fusion partner complexes also contain unique components. For instance, AF9 co-purifies with transcription factor IID (TFIID) subunits [225], AF4 and AFF4 co-purify with the transcriptional coactivator complex Mediator [225,237] and AF9 and ENL directly interact with CBX8 [223,225,238,239,240]. While it is not yet clear if the interactions with Mediator and TFIID contribute to MLL-AF4 or MLL-AF9 leukemogenesis, CBX8 has been recently shown to be essential for MLL-AF9 induced leukemogenesis, potentially by recruiting the histone acetyltransferase Tip60 [240].

MLL-FPs contain truncated versions of the various fusion partners and thus they retain most, but not always all, of the fusion partner protein interactions. For example, in the MLL-AF4 fusion the *N* terminus of AF4, which is responsible for the direct interaction with pTEFb, is missing [227]. However, since the AF4-C portion of the protein can heterodimerize with AFF4 (and weakly homodimerize with AF4), the MLL-AF4 protein is still able to indirectly interact with the pTEFb complex (Figure 5Aiii). The N termini of the AF9 and ENL proteins are missing in MLL-AF9 and MLL-ENL fusions and thus are missing the PAF1C interaction domain [232]. However, all MLL-FPs still interact with PAF1C through the CXXC domain [69,78].

Considering the presence of unique protein interactions and the fact that many fusion partner protein-protein interactions are mutually exclusive [225,227,232,234], it seems likely that MLL-FPs participate in multiple distinct complexes in the cell (Figure 5). For example, based on exclusivity of some of the factors in wild type fusion partner complexes, it seems likely that MLL-AF4 could exist in three distinct mutually exclusive complexes containing AF9, ENL or AFF4/pTEFb/ELL/EAF (Figure 5Ai–iii). Conversely, MLL-AF9 or MLL-ENL could be present in either a DOT1L or an AFF4/pTEFb/ELL/EAF containing complex, but not both (Figure 5Bi,ii). Currently, it is unclear if AF9/ENL and CBX8 or PAF1C interactions are compatible with other protein interactions, but this could increase the number of possible subcomplexes in the cell for different MLL-FPs. Taken as a whole, the protein interaction data suggests that different MLL-FPs exist in at least some unique protein complexes (Figure 5), and this could have distinct functional outputs at regulatory targets, perhaps explaining at least some of the phenotypic differences between different MLL-FPs. Below we explore these possibilities.

## 8. A Unifying Molecular Model for the Six Common MLL-FPs?

What is the functional significance of all these interactions? The current model of MLL-FP leukemogenesis suggests BRD4 binds to acetyl lysine residues on H3 and H4, and then recruits PAF1, MLL-FPs and the SEC (via an interaction with pTEFb and through the MLL-FPs themselves) to a subset of important target genes (including *MYC*, *MYB* and *BCL2*) causing increased gene expression [69,78,185,186,241,242]. Since the six common fusion partner proteins are components of the same web of protein interactions, these MLL-FPs could recruit the same SEC to regulatory targets *in vivo*. ChIP experiments seem to support this possibility since most of the components of the SEC are present at the same gene targets, even in different MLL-FP leukemias [225,226,227,230,231]. This simple, unifying mechanism is also supported by the observation that highly specific inhibitors that target the acetyl binding pocket of BRD4 disrupt activation of some MLL-FP target genes such as *MYC*, and impair the growth of a wide range of different MLL-FP-mediated leukemias [185,186].

However, even among these six common FPs, a simple unifying model of MLL-FP molecular activity does not fit the complexity of the interaction data (see above) or the fact that individual MLL-FPs cause different leukemias that are determined by the fusion partner itself. This instead suggests that the specific fusion partners in particular MLL-FPs have different functions and thus don’t act through a completely common molecular mechanism. In addition, even though the AF6 protein doesn't interact with any components of the SEC, ChIP experiments indicate that in MLL-AF6 leukemias, all the same factors are bound to gene targets as in MLL-AF4, MLL-ENL and MLL-AF9 leukemias [227]. MLL-AF6 is thought to function through dimerization [243], indicating that SEC recruitment/activity could be controlled indirectly through normal gene activation [231]. Although we have focused our discussion on the six most common MLL-FPs, it is also worth noting that there are at least 59 other gene partners that can cause MLL-FP leukemias and none have been implicated in SEC interactions, but it is possible that they are all able to recruit the SEC indirectly in a way that is similar to MLL-AF6. Thus it seems unlikely that the major role of MLL-AF9, MLL-AF4, MLL-ENL, MLL-AF10 and MLL-ELL is to simply recruit the rest of the SEC. A full understanding of the molecular mechanisms of MLL-FP leukemogenesis requires detailed individual information on each component of the MLL-FP interactome as well the function of the individual proteins in the context of larger complexes. A detailed discussion of key MLL-FP complex components is outlined below.

## 9. Epigenetic and Transcriptional Mechanisms of the MLL-FP Interactome

Certain individual components of the MLL-FP interactome are well studied on the molecular level. Recent work has suggested that at the bulk of promoters, RNA polymerase II (Pol II) is paused proximal to the promoter [27,244,245,246,247,248]. Relief of proximal promoter pausing and full activation of gene expression is a highly regulated process that requires the activity of several components of wild type SEC complexes. CDK9, along with Cyclin T1 or Cyclin T2, is part of the pTEFb complex and is required for the phosphorylation of serine 2 on the Pol II C-terminal domain [249,250,251,252,253]. Serine 2 phosphorylation disrupts binding of the repressive DSIF/NELF (aka suppressor of Ty 5 homolog or SUPT5H and negative elongation factor) complex and helps Pol II transition to an actively elongating form [27,251,252], a process that is stabilized by a BRD4-pTEFb interaction [241,242]. Once Pol II is converted to an actively elongating form, elongation factors such as ELL and ELL2 are able to increase the rate of Pol II transcription [254,255], by maintaining alignment of the 3'-OH terminus of the nascent transcript and thus preventing Pol II backtracking [256]. Recent work also suggests that ELL containing complexes are recruited to promoters through an interaction between EAF1/2 and the Mediator subunit Med26, providing a potential link between transcription initiation events and the transition to productive elongation [237]. Additional factors such as AF4 and/or AFF4 could potentially act in concert with ELL/2 to increase transcription elongation at target genes *in vivo*. However, the ELL containing AF4 complex has no apparent additional elongation activity *in vitro* relative to purified ELL/EAF components [225]. One possibility is that, as with the elongation factors PAF1C and SII [162], the AF4/AFF4 enhancement of transcription elongation may only be apparent in the presence of nucleosomes. A full answer to this question awaits testing the transcription activity of these purified complexes on a chromatin template.

Whatever the specific activities of the AF4 or AFF4 proteins might be, AFF4 stabilizes ELL protein levels [226] and overexpression of AFF4 prevents the (E3 ubiquitin ligase) seven in absentia homolog 1 (SIAH1) mediated degradation of ELL2 [234]. Early work also identified an interaction between AF4 and SIAH1/2 which promotes rapid degradation of the wild type AF4 protein [257,258], but the work of Liu *et al*. suggests that ELL2 is the primary target of SIAH1 mediated degradation, and that AF4/AFF4 mediated degradation is a secondary event [234]. MLL-FPs lack the PHD fingers of MLL and are resistant to normal MLL degradation pathways [85,151,259], suggesting that one possible function of the MLL-AF4 fusion protein is to stabilize ELL or ELL2 containing SEC complexes. Interestingly, interaction of the AF4-MLL fusion protein with MLL-C prevents SIAH mediated degradation of AF4-MLL [257], suggesting that the AF4-MLL fusion may also be involved in stabilizing ELL and ELL2 containing SEC complexes.

The six components of the PAF1 complex produce robust enhancement of transcription on a chromatin template [162], and thus may also contribute directly to increased transcription of MLL-FP target genes. Initial experiments analyzing the role of PAF1C in MLL-FP leukemogenesis focused on its role in recruiting MLL-FP complexes to specific gene targets [69,78]. However, it also remains possible that MLL-FPs enhance the activity/stability of PAF1C in some way. Little is known about the specific molecular functions of the AF9 and ENL proteins, but they directly interact with PAF1C and may help stabilize and/or recruit PAF1C as well as other components of the SEC [232]. The MLL-AF4 protein retains the ability to interact with wild type ENL and AF9 (Figure 5 Ai,ii) suggesting that MLL-AF4/ENL/AF9 containing complexes could have specific effects on PAF1C activity at target genes.

Although DOT1L containing complexes appear to be distinct from the rest of the SEC, inhibition of the H3K79 methyltransferase activity of DOT1L disrupts MLL-FP leukemogenesis [188,220,260,261,262,263], and increased H3K79Me levels are associated with multiple MLL-FPs including MLL-AF9, MLL-ENL and MLL-AF4 [12,13,230]. Although H3K79Me is associated with productive transcription elongation [27,28] and has connections with PAF1C mediated transcription elongation [264], no readers of H3K79Me2/3 have been identified and little is known about what specific role this histone mark has in transcription. However, enzymatically dead versions of DOT1L are unable to rescue DOT1L deficiencies in MLL-FP leukemias [263] and DOT1L enzymatic inhibitors have little effect on other leukemias [261], suggesting that whatever the specific role is, H3K79Me2 is key for maintaining the expression of MLL-FP target genes.

Menin and LEDGF are both key contributors to MLL-FP leukemogenesis [86,87,88,91,117] and they were initially thought to be essential for the recruitment of wild type MLL and MLL-FPs to gene targets *in vivo* [90,91,115,117,118,119]. However, since Menin (and by extension LEDGF/p75) are components of both MLL and MLL2 complexes [68,92], Menin binding to target genes is partially dependent on MLL-ENL binding [117], and the major Menin and LEDGF/p75 interaction sites on MLL-FPs are dispensable for MLL-FP recruitment to the *HOXA9* locus [69], the main function of these proteins is not likely to be recruitment.

Interestingly, the Menin-MLL interaction creates a binding pocket for LEDGF such that LEDGF can only interact with MLL and Menin as a trimeric complex [90]. The PWWP domain of LEDGF is essential for leukemogenesis [91] and binds to H3K36Me [265], but it also directly contributes to transcription activation [266,267], in part by controlling splicing at specific target genes [265]. Together, this data suggests that the main role of the MLL-Menin interaction is to recruit LEDGF, which then may help promote transcription and splicing at key target genes.

## 10. Therapeutic Inhibitors of MLL-FP Leukemias

Several recent excellent reviews have covered the topic of epigenetic inhibitors in general and MLL small molecule inhibitors in particular [16,268,269,270,271,272,273] and as such we will only briefly deal with this topic here by highlighting a few key developments. A survey of histone modifications initially identified H3K79Me2 as a mark associated with MLL-ENL and MLL-AF9 activation of target genes [13], but the first study to directly implicate DOT1L in MLL-FP leukemias was the work of Okada *et al.* [220]. As already mentioned, more recent work has shown that DOT1L is key in maintaining MLL-FP leukemogenesis [188,260,262,263] and it is specifically the KMT (lysine methyltransferase) activity of DOT1L that is required [261,263]. In early experiments, a highly specific DOT1L KMT inhibitor appears to disrupt the growth of MLL-FP leukemias [261]. The development of more potent DOT1L inhibitors [274] and a more detailed analysis of the general toxicity of these inhibitors will be key in determining their efficacy in clinical trials, but this validates the approach of targeting specific writer domains as an effective way of disrupting leukemogenesis.

Using a retroviral transduction assay, an analysis of multiple MLL-FP leukemias revealed overexpression of the H3K4/K9 eraser KDM1A (lysine (K)-specific demethylase 1A) [130,275,276]. Interestingly, KDM1A expression is required for the maintenance of MLL-FP leukemias and treatment of MLL-FPs with KDM1A inhibitors had a profound effect on leukemic growth *in vitro *as well as *in vivo* [275]. Although the inhibitors used in this study displayed some hematopoietic cell toxicity [275], KDM1A represents another example of targeting the enzymatic activity of a protein with specific inhibitors.

Recent exciting work has also identified highly specific inhibitors of the BET family of Bromodomain containing proteins (including BRD4) as effective inhibitors of a range of different leukemias, including those containing MLL-FPs [185,186,277,278,279]. Initially considered to work primarily through inhibition of BRD4 mediated MYC gene expression, the molecular mechanism is likely to be more complex than this as MYC expression cannot completely rescue the effect of the BET inhibitor JQ1 [186] and some leukemias that express MYC are not sensitive to JQ1 [186,279]. The complete molecular mechanisms of BET inhibition remain to be elucidated, but the discovery of this potent class of inhibitors validates the targeting of reader domains as an effective way of disrupting leukemias and potentially other cancers.

The above examples provide evidence that specifically targeting readers, writers and erasers may be an effective way of developing small molecule inhibitors of MLL-FP leukemias. In a different approach, work in the Cierpicki lab specifically focused on structural studies of the MLL-Menin interaction as a way of developing inhibitors of this important MLL protein complex component [87,89]. Early work by Yokoyama *et al.* showed that Menin was essential for MLL-FP leukemogenesis [86] and Grembecka *et al*. used Menin-MLL structural data to develop specific Menin-MLL small molecule inhibitors that disrupt the growth of MLL-FP leukemias [88]. MLL-FP cells appear to be highly sensitive to these inhibitors and it will be interesting to see how effective they are in animal model systems and potentially clinical trials.

Other examples of inhibitors that appear to disrupt the growth of MLL-FPs (reviewed in [268]) include pTEFb inhibitors [224], an AF4-AF9 interaction inhibitor [217,218,280], GSK-3 inhibitors [281] and FLT3 inhibitors [282]. In the future, the fact that wild type MLL is essential for MLL-FP activity [69,119], suggests that inhibitors of WDR5, RBBP5 or the MLL SET domain may also prove to be effective therapeutic agents. However, since MLL is essential for normal hematopoiesis such inhibitors may have undesirable side effects. Instead, the DNA binding capability of the MLL CXXC domain is essential for MLL-FP leukemogenesis [67,69,76,283], but this activity appears to be less important in the wild type MLL protein [69]. This suggests that inhibitors designed to target the DNA binding activity of the CXXC domain may specifically disrupt MLL-FP proteins without disrupting the activity of the wild type MLL protein, thus potentially proving to have reduced cellular toxicity. Several downstream target genes of MLL-FPs are essential for leukemogenesis including *MYB* [182] and *HOXA9* [171,172], but specifically inhibiting the activity of these transcription factors with small molecules is likely to prove challenging. Further analysis of downstream pathways controlled by MLL-FPs could instead reveal the existence of novel readers, writers or erasers that have functional domains that can be targeted in similar ways as the examples above. The possibility that leukemias could be highly individualized suggests that effective therapies may require a cocktail of different inhibitors, and thus development in this area is likely to continue.

## 11. Conclusions

Although MLL-FP leukemias appear to be relatively simple from a genetic point of view, this masks a great deal of complexity on the protein, gene expression and epigenetic levels. What can we make of all this complexity and the unique phenotypic outputs of different MLL-FPs? Different MLL-FPs may promote different transcriptional profiles by assembling and stabilizing unique complexes, potentially at unique regulatory targets. Slight differences between MLL-FPs on the transcriptional level could produce profound differences on the protein or miRNA levels. Thus future proteomic [284] and miRNA studies may need to treat different MLL-FPs as separate leukemia classes. Epigenetic inhibitors that were initially developed by studying MLL-FP leukemias [14,15,16] have an impact on a much wider range of different cancers [277,279], suggesting MLL-FP leukemias may provide a useful model for studying the role of epigenetics in human disease in general.
